# Intracardiac Echocardiography Guided Transeptal Catheter Injection of Microspheres for Assessment of Cerebral Microcirculation in Experimental Models

**DOI:** 10.1155/2013/595838

**Published:** 2013-09-11

**Authors:** Judith Bellapart, Kimble R. Dunster, Sara Diab, David G. Platts, Christopher Raffel, Levon Gabrielian, Marc O. Maybauer, Adrian Barnett, Robert James Boots, John F. Fraser

**Affiliations:** ^1^Intensive Care Department, Royal Brisbane and Women's Hospital, Burns-Trauma & Critical Care Research Centre, The University of Queensland, Herston, QLD 4025, Australia; ^2^Medical Engineering Research Facility, Queensland University of Technology, QLD, Australia; ^3^Department of Cardiology, The Prince Charles Hospital, QLD, Australia; ^4^Medical School Research Centre, Adelaide, SA, Australia; ^5^Department of Anesthesiology, University of Texas Medical Branch, Galveston, USA; ^6^Intensive Care Department, The Prince Charles Hospital and Medical Engineering Research Facility, Queensland University of Technology, QLD, Australia; ^7^Department of Anaesthesiology and Intensive Care, Philipps University, Marburg, Germany; ^8^Institute of Health and Biomedical Innovation & School of Public Health and Social Work, Queensland University of Technology, Australia

## Abstract

The use of microspheres for the determination of regional microvascular blood flow (RMBF) has previously used different approaches. This study presents for the first time the intracardiac injection of microspheres using transeptal puncture under intracardiac echocardiography guidance. Five Merino sheep were instrumented and cardiovascularly supported according to local guidelines. Two catheter sheaths into the internal jugular vein facilitated the introduction of an intracardiac probe and transeptal catheter, respectively. Five million colour coded microspheres were injected into the left atrium via this catheter. After euthanasia the brain was used as proof of principle and the endpoint for determination of microcirculation at different time points. Homogeneous allocation of microspheres to different regions of the brain was found over time. Alternate slices from both hemispheres showed the following flow ranges: for slice 02; 0.57–1.02 mL/min/g, slice 04; 0.45–1.42 mL/min/g, slice 06; 0.35–1.87 mL/min/g, slice 08; 0.46–1.77 mL/min/g, slice 10; 0.34–1.28 mL/min/g. A mixed effect regression model demonstrated that the confidence interval did include zero suggesting that the apparent variability intra- and intersubject was not statistically significant, supporting the stability and reproducibility of the injection technique. This study demonstrates the feasibility of the transeptal injection of microspheres, showing a homogeneous distribution of blood flow through the brain unchanged over time and has established a new interventional model for the measurement of RMBF in ovine models.

## 1. Introduction 

The measurement of microcirculation in specific organs has been the focus of multiple studies since 1967, when 50 *μ* diameter radionuclide-labelled carbonised spheres were injected in the foetal umbilical vein of sheep [1]. Since then, several studies have introduced modifications to the methods including the description of the reference sample for the calculation of blood flow [2], the injection of microspheres into the left atrium (LA) to minimise peripheral and central shunting [3], transient occlusion of the pulmonary artery also to minimise shunting [4], and the use of two different radioactive spheres and simultaneous LA and right atrium (RA) injections (*via a left thoracotomy*) to discriminate shunting from pulmonary blood flow [5]. Substantial modifications in this technique also included the use of nonradioactive spheres [6] which added significant environmental and logistical advantages. In addition, the use of smaller size spheres showed to be less detrimental to end-organ circulation [7] minimising the obstructive component of spheres when circulating through small capillaries, therefore, facilitating their cytometric count. Microspheres have not been previously injected using a transeptal approach.

Transeptal catheterization refers to the intracardiac puncture of the interatrium septum with the intention of accessing the LA from the RA. This technique was originally developed by Dr. John Ross in 1957, at the National Institute of Health, Maryland, USA [8]. The targeted area in a transeptal catheterization is the Fossa Ovalis (FO), as it is the thinnest region within the septum. Guidance of the procedure as well as identification of the FO has been facilitated by the use of both fluoroscopy [9] and ultrasound [10]. Transesophageal echocardiography (TEE) is superior to transthoracic (TTE) due to improved visualisation of the FO and its “*tenting*” by the tip of the catheter, therefore, confirming its correct positioning [11]. Recent modalities such as intracardiac echocardiography (ICE) (AcuNav probe, Medical Systems, Mountain View, CA) have improved spatial resolution [9].

Although there is no randomised controlled trial (RCT) demonstrating the superiority of a particular ultrasound guided transeptal technique, several series of ICE guided transeptal punctures [12, 13] have shown its feasibility and safety. The aim of this study was to develop an ovine model of intracardiac echocardiographic guided transeptal catheterization for the injection of colour-coded microspheres into the LA as a vehicle to assess cerebral blood flow.

## 2. Materials and Methods

### 2.1. Animal Care and Preparation

All experimental procedures were approved by the Animal Ethics Committee of the Queensland University of Technology.

A convenience sample of five Merino ewes, weighing 65 ± 6.01 kg, was transported from an approved animal farm and housed for three days at the medical engineering research facility, in large grassed yards according to local ethics committee requirements. Sheep were monitored daily to assess their nutritional and comfort state and their levels of stress.

Animal instrumentation included inserting a triple lumen central line (Cook Medical INC., QLD, Australia) and two 16Fr introducer sheaths in the right internal jugular (RIJ) vein, under local anaesthesia with 50 mg lignocaine, and physical animal restrain. Via the central line, general anaesthesia was administered using ketamine bolus of 5 mg/kg followed by a maintenance infusion of 0.5–1 mg/kg/h. Midazolam by infusion was given at a rate of 0.5 mg/kg/h in addition to buprenorphine at 0.01 mg/kg/h. Hydration was maintained with Hartmann's solution initially at 2 mL/kg/h and titrated to maintain a central venous pressure (CVP) of 6–10 mmHg. To avoid intraatrium codependency, a pulmonary artery catheter (PAC) was not used as reported in previous models [14]. The right atrium is potentially crowded by both the ICE probe and transeptal catheter. Addition of a PAC inside this small area impairs the transeptal technique. Orotracheal intubation used a size 10 mm endotracheal tube* (SIMS Portex, UK)*. Under anaesthesia, the sheep were mechanically ventilated with tidal volumes of 8 mL/kg, a rate of 12 breaths per minute, PEEP 5 cm H_2_O, and initial fractional inspired oxygen (FiO_2_) of 1.0 titrated to maintain partial pressure of oxygen (PaO_2_) of >95 mmHg. The instrumentation phase followed with the insertion of a femoral arterial catheter via Seldinger technique to ensure continuous blood pressure monitoring.

During deep anaesthesia when sheep were instrumented and haemodynamically stable a transeptal catheter was passed into the left atrium under ICE surveillance by experienced interventional cardiologists. 

### 2.2. Protocol for the Transeptal Catheterization and Injection of Microspheres

**Figure 1:**
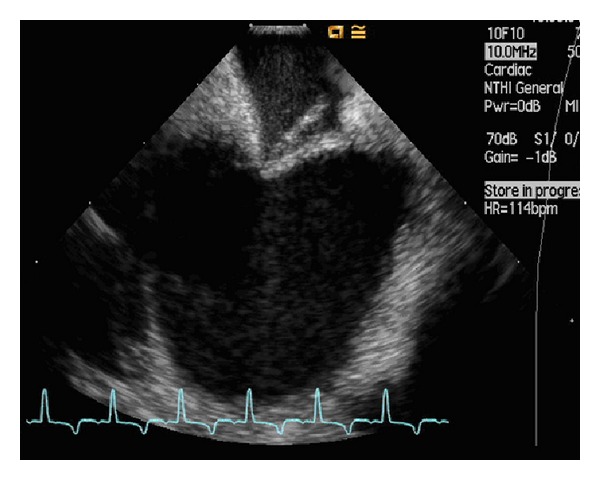
Intracardiac echocardiography (ICE) image of the interatrium septum demonstrating bowing of the midseptum by the Brockenbrough needle. Tenting of the interatrium septum by the Brockenbrough needle is seen, using a 10 MHerz ICE probe. Subject's heart rate of 140 beats per minute without any arrhythmic response to this intervention.

**Figure 2:**
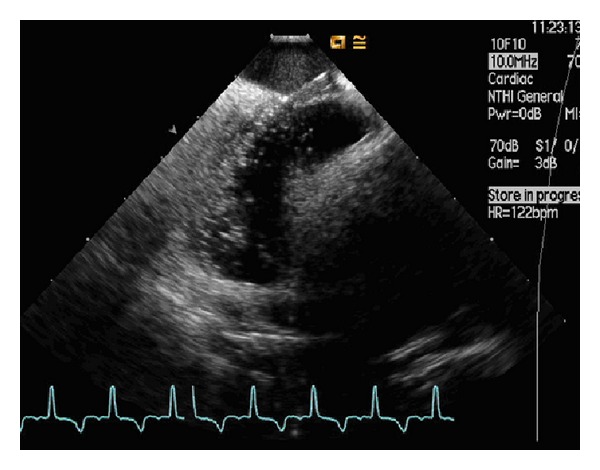
Agitated saline bubble study during intracardiac echocardiography. Agitated saline bubbles are shown into the LA confirming successful septal puncture. Subject's heart rate and rhythm are unchanged, indicating good tolerance to this manoeuvre.

**Figure 3:**
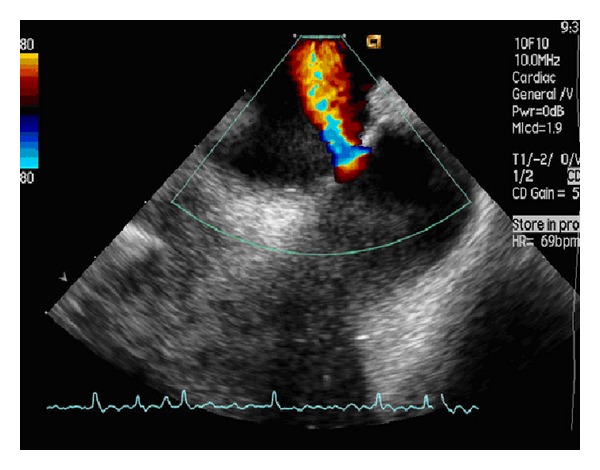
Colour Doppler imaging during intracardiac echocardiography. Flow through interatrium septum is seen via Doppler imaging, excluding iatrogenic perforation of atrium wall and confirming correct positioning of transseptal catheter into the LA.

Two 11F Terumo sheaths located in the RIJ allowed the insertion of the intracardiac ultrasound probe (Acuson AcuNav probe, CA) and the transeptal catheter (Mullins TS introducer, Medtronic), respectively. Echocardiography images were obtained using an Acuson Sequoia C512 scanner (Siemens, CA). After visualisation of the interatrium septum by the ICE probe from the RA, the curved catheter delivering a dilator (*which straighten the catheter's tip*) was advanced adjacent to the probe. With the tip of the catheter pointing at the FO, a needle (Brockenbrough needle, Medtronic) (Figure 1) was introduced through the dilator, keeping its tip within the dilator's lumen; it is in this position that the assembly was advanced through the FO and the needle protruded into the LA. Over a guidewire a pig-tail catheter was passed into the LA with its position confirmed by ICE using an injection of agitated saline into the LA (Figure 2) without retrograde leaking or without perforation. A Doppler signal revealed the confirmation of flow through the septum only, ensuring the absence of side-wall perforation (Figure 3).

### 2.3. Protocol for Microspheres Injection

After insertion of the transeptal catheter and confirmation of its correct positioning, hourly injection of colour-coded microspheres (E-Z TRAC; Interactive Medical Technology, Los Angeles, CA) used the following protocol.

A thorough mixture of the content of each colour microspheres vial and an extraction of 0.8 mL volume containing 5 million spheres was followed by the flushing of the spheres dilution into the transeptal catheter line. Commencement of the withdrawal pump at a rate of 10 mL/min. was followed by the injection of the microspheres over 10 seconds, 30 seconds after the commencement of the withdrawal pump. Discontinuation of the withdrawal pumps two minutes after commencement followed by the withdrawal of the pump to allow the Tween 80 reagent to clear the femoral tubing and to dilute microspheres as previously described [15]. 

### 2.4. Aspects of the Injection of Colour-Coded Microspheres

Approximately 5 million colour-coded spheres of 15 micron diameter each were injected at each hour, using a 1 mL syringe and a saline flush following each injection. Randomly assigned colours were attributed to each injection time in order to minimise selection biases, as previously described [14, 16]. For five hourly time points, five different colours were chosen with the intention to track RMBF at each time point during cytometric counting. Microspheres had been preserved at room temperature and not exposed to sun light, heating, or vibration as advised by the manufacturer. Following recommendations, the mixing of these microspheres was performed using a manual technique in order to minimise foaming and, therefore, unequal concentration of spheres in each injection.

### 2.5. Euthanasia and Postmortem Tissue Manipulation

After five hours of continuous monitoring and microspheres injection, sheep were euthanized under nonrecovered anaesthesia with a bolus injection of 0.5 mL/kg of sodium pentobarbitone (Lethabarb). After confirmation of death, craniotomy for brain harvesting and brain weighing were followed by fixation in 10% formalin for three weeks. Samples from skin, gut, and heart were extracted from the last sheep to demonstrate systemic distribution of microspheres. Brains were sectioned in 5 mm slices and numerically labelled in an anteroposterior direction. An arbitrary sectioning of the brain was designed after consensus from all clinical investigators, with only *even*-numbered slices processed for cytometric count. 

### 2.6. Quantification of Microvascular Blood Flow

Cytometric count of microspheres is a standard process widely used [14, 17, 18] and validated. Cytometric count of spheres allows calculation of RMBF through a process that includes the known microspheres concentration injected into the arterial supply of an organ and a “reference sample of blood” (or arterial blood sample withdrawn at a known rate during a known length of time) [19]. This way, RMBF will be proportional to the number of microspheres trapped in the targeted tissue in relation to the total quantity of spheres per mL of blood per minute of the reference sample and corresponds to the equation [20]

(1) RMBF  (mL/min⁡/g)=(Total  tissue  spheres)[(Tissue  weight,g)×(Reference(Spheres/mL))/min⁡].

### 2.7. Statistical Analysis

Cytometric counts for each time and slice were averaged. Means for each time and for the difference between each time point from baseline were also calculated. To test for any statistically significant differences, the differences from baseline were regressed against time (as a categorical variable) using linear regression with time as the independent variable. These regressions were performed for each sheep. All analysis was performed using the R software (http://www.r-project.org/). We visually examined the results using plots for each sheep over time. To test statistical significance of the differences seen, we used mixed effects regression model with a random intercept for each sheep to control for repeated responses from the same sheep. The plots and regression models were created using R version 3.0.0.

## 3. Results

The results of this study demonstrate that colour-coded microspheres, injected into the LA using this method, systemically distribute to multiple areas within the brain as well as to other organs such as heart and skin, over time.

Alternate slices from postmortem brains showed the following RMBF range: Slice 02; 0.57–1.02 mL/min/g, slice 04; 0.45–1.42 mL/min/g, slice 06; 0.35–1.87 mL/min/g, slice 08; 0.46–1.77 mL/min/g, slice 10; 0.34–1.28 mL/min/g, slice 12; 0.35–1.58 mL/min/g, slice 14; 0.29–1.15 mL/min/g. RMBF measurements within the midbrain showed a range of 0.45–0.76 mL/min/g. RMBF in the pons showed a range of 0.64–2.28 mL/min/g. RMBF at the medulla showed a range of 0.66–4.8 mL/min/g (Table 1). The stability of the catheter's placement into the LA as well as that of the subject's cardiovascular function was reflected by a homogenous RMBF among different slices and over time (Tables 2 and 3). 

An exemption was found with subject number two, as nondetectable amount of spheres were found during times 1 to 4 was due to a nondetectable amount of microspheres in the referral sample (blood at the descendant aorta) interfering in the estimation of RMBF.

**Figure 4:**
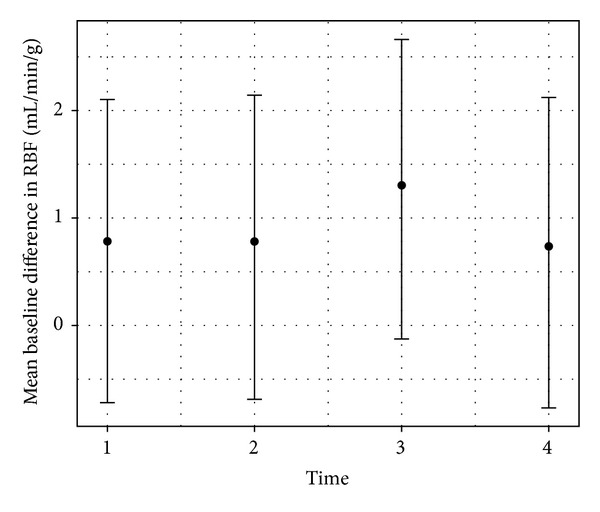
Means (dots) and 95% confidence intervals (vertical lines) of RMBF by time. Estimates from a mixed model analysis. Results where the confidence interval includes zero are statistically not significant.

To assess the significance of the apparent variability of the RMBF at different times with respect of their baseline time, estimates from a mixed model showed that the confidence interval of the mean baseline difference in RMBF over time points does include zero which means that despite the visual variability, this is not statistically significant (Table 4). All RMBF times were higher than baseline on average, but none was statistically significant at the 5% level as each 95% confidence interval includes zero (Figure 4).

**Figure 5:**
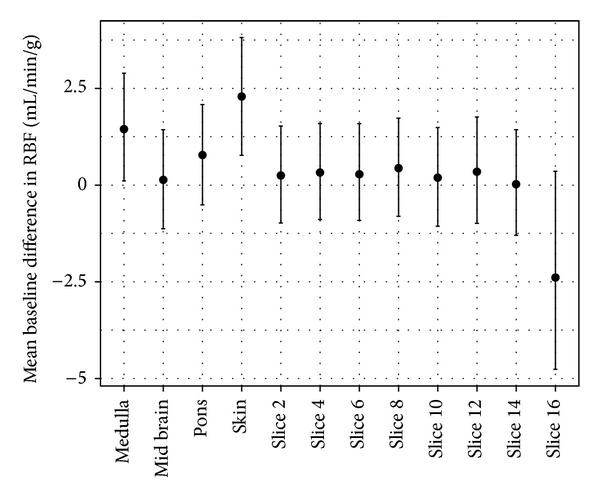
Means (dots) and 95% confidence interval (vertical lines) by slice. Estimates from mixed-model analysis. Results where the confidence interval includes zero are statistically not significant.

To assess the significance of the apparent variability of RMBF throughout all slices, estimates from a mixed model showed that the confidence interval of the mean difference in RMBF throughout all slices does include zero which means that despite the visual variability, this is not statistically significant (Table 5). All slices except for medulla, skin, and slice number 16 had similar RMBF values, although the confidence interval of medulla and slice 16 also included zero implying that variability was statistically not significant. Instead, only skin had a statistically significant variability of RMBF over time (Figure 5).

**Figure 6:**
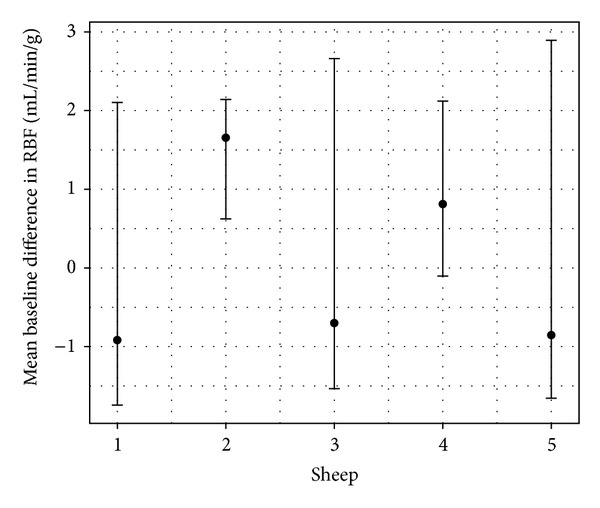
Means (dots) and 95% confidence intervals (vertical lines) by sheep. Estimates from mixed-model analysis. Results where the confidence interval includes zero are statistically not significant.

Also, to assess the significance of the apparent variability of RMBF throughout all subjects, estimates from a mixed model showed that the confidence interval of the mean baseline difference in RMBF amongst subjects also include zero which means that despite the visual variability, this is not statistically significant (Figure 6).

To demonstrate that microspheres were also distributed systemically into other organs, slices were analysed from myocardium and abdominal skin. Mean RMBF into myocardium was 0.02–0.07 mL/min/g whereas mean RMBF in skin was 1.2–4.07 mL/min/g. 

## 4. Discussion

This study demonstrates for the first time, the feasibility of the transeptal catheterization of the LA for the administration of colour-coded microspheres, in an experimental ovine model. We have demonstrated that transeptal puncture is a safe technique with uncommon complications. The few complications found during this procedure were mainly related to codependency between the ICE probe and the transeptal catheter inside the small area of the RA. None of the sheep experienced ventricular wall rupture or iatrogenic trauma to the tricuspid valve apparatus. Transient supraventricular arrhythmias were seen in two of the five subjects during the transeptal puncture all resolving spontaneously. 

The main aim of this procedure was to inject microspheres directly into the LA allowing intracavitary mixing prior to their systemic ejection. The central mixing of microspheres is required to ensure homogeneous distribution of spheres in each stroke volume. Our method differs from the studies that have previously used direct LV injection through retrograde canulation [21]. Also, avoiding retrograde canulation of the aorta minimises chaotic flow at the aortic root, therefore, enhancing systemic distribution of spheres in a homogeneous fashion.

The stability of the catheter into the LA after its insertion is essential to complete each study. In our study, all subjects maintained the catheter *in situ* through the length of each study day, demonstrated by a direct visualisation of the tip of the catheter inside the LA, via a left thoracotomy, during the postmortem phase. This is a relevant factor in this experimental method as an incidental removal or shift of the catheter's tip outside of the LA would imply the cessation of the study. Considering how regularly microspheres were injected, in case of misplacement there would have been no sufficient time for a relocation of the catheter. The stability of the injection site is demonstrated by the finding of a similar distribution of microspheres within multiple tissues from baseline (Tables 2 and 3) which are of similar magnitude than in other studies [22]. The plausible explanation to the nondetectable spheres in times 1 to 4 for subject number two is the presence of a retrograde flush of microspheres into the RA simultaneously with each injection for times 1 to 4. This effect could be related to the dilation of the insertion site at the FO, during the transeptal procedure, leading to a shunt of microspheres through the intrapulmonary circulation and leading to a nondetectable amount of microspheres at the systemic circulation in a delayed phase. The other plausible explanation could be related to the presence of LA thrombus coating with microspheres as injections is performed and therefore, reducing the total amount of microspheres distributed systemically with each stroke volume.

As demonstrated before [1], the use of microspheres for the study of microcirculation has shown several advantages. First, spheres do not recirculate after injection. Second, spheres' distribution is directly related to the magnitude of blood flow in each organ. Third, the injection of microspheres does not modify inner-cardiovascular properties of the subject. These properties offer the benefit of selectively assessing small vascular networks such as the brain. In this study, a homogeneous distribution of microspheres was demonstrated in all sections of the sheep brain as well as within other extracranial organs. Also, systemic distribution of microspheres was sustained over time, demonstrating that for each injection time, RMBF was maintained stable. An exemption was found within the skin RMBF (Figure 5), as a significant variability was demonstrated with its confidence interval of the mean baseline difference in RMBF not including zero. A likely explanation lies upon the known physiological variability of the skin perfusion. The results of this study are in concordance with previous studies using microspheres injection via left thoracotomy [14, 18] demonstrating the reliability of this novel approach.

Currently the most extensive information available regarding cerebral microcirculation is based on experimental models of burns after fluid resuscitation [23], in the setting of septic shock [18] and inhalational injury using injection of microspheres into the LA through a left thoracotomy [14, 24, 25]. Few studies have assessed cerebral microcirculation after traumatic head injury [26, 27] using the same approach. In view of this methodological background, our study offers two main advantages. First, regarding procedural aspects of the microspheres technique, a direct injection of coloured spheres into the LA through a transeptal puncture allows homogeneous mixing of the spheres and reliable systemic distribution with each stroke volume. Second, an unnecessary thoracotomy adds an aspect of noninvasiveness and safety to this procedure. Also, the absence of a surgical recovery downtime leads to shorter instrumentation phase, shorter exposure for the animal models, and lesser maintenance costs. 

## 5. Conclusion

Left atrium injection of colour-coded microspheres using ICE technology is a feasible approach for the analysis of cerebral microvascular blood flow. The reproducibility and homogeneous distribution of spheres with this technique, as well as the stability of the microspheres distribution over time, leads to the establishment of this approach as a new interventional model for the measurement of RMBF in ovine models.

## Supplementary Material

Figure 1: Results of RMBF over time by sheep and slice.
Figure 2: Differences from baseline over time by sheep and slice.
Figure 3: Differences from baseline over time by sheep and tissue.

## Figures and Tables

**Table 1:** RMBF means (standard deviations) by each brain slice over time (*T*), expressed in mL/min/g.

Slice	*T*0	*T*1	*T*2	*T*3	*T*4
Heart	0.05 (NA)	0.06 (NA)	0.05 (NA)	0.02 (NA)	0.07 (NA)
Skin	2.70 (NA)	1.65 (NA)	2.38 (NA)	1.26 (NA)	4.07 (NA)
Medulla	1.06 (0.71)	0.74 (0.57)	1.92 (2.06)	4.83 (6.54)	0.66 (NA)
Mid Brain	0.45 (0.24)	0.58 (0.35)	0.55 (0.42)	0.76 (0.74)	0.63 (0.33)
Pons	0.64 (0.21)	0.99 (0.90)	0.86 (0.83)	2.28 (3.20)	0.79 (0.27)
Slice 02	0.57 (0.26)	1.02 (1.20)	0.89 (1.13)	NA (NA)	0.71 (0.47)
Slice 04	0.45 (0.15)	0.71 (0.59)	0.76 (0.88)	1.42 (1.70)	1.04 (1.11)
Slice 06	NA (NA)	0.67 (0.55)	0.72 (0.82)	1.87 (3.00)	0.35 (0.33)
Slice 08	0.52 (0.30)	0.63 (0.41)	0.46 (0.33)	1.77 (2.85)	1.46 (1.93)
Slice 10	0.42 (0.16)	0.45 (0.27)	0.34 (0.21)	1.28 (2.12)	0.38 (0.33)
Slice 12	0.66 (0.26)	0.75 (0.67)	0.35 (0.35)	1.58 (2.28)	0.86 (0.67)
Slice 14	0.48 (0.13)	0.67 (0.58)	0.31 (0.30)	1.15 (1.58)	0.29 (0.29)

**Table 2:** Table of RMBF mean differences (standard deviations) from baseline by slice and time.

Slice	*T*1 − *T*0	*T*2 − *T*0	*T*3 − *T*0	*T*4 − *T*0
Heart	0.01 (NA)	0 (NA)	−0.02 (NA)	0.02 (NA)
Medulla	−0.33 (0.57)	0.86 (2.1)	3.8 (6.5)	0.1 (NA)
Mid Brain	0.13 (0.35)	0.1 (0.42)	0.31 (0.74)	0.06 (0.33)
Pons	0.36 (0.9)	0.23 (0.83)	1.6 (3.2)	0.04 (0.27)
Skin	−1.0 (NA)	−0.32 (NA)	−1.4 (NA)	1.4 (NA)
Slice 02	0.4 (1.2)	0.27 (1.1)	2.4 (5.5)	0.22 (0.47)
Slice 04	0.26 (0.59)	0.31 (0.88)	0.97 (1.7)	0.55 (1.1)
Slice 06	0.12 (0.55)	0.17 (0.82)	1.4 (3)	−0.02 (0.33)
Slice 08	0.21 (0.41)	0.05 (0.33)	1.2 (2.8)	0.87 (1.9)
Slice 10	−0.02 (0.27)	−0.14 (0.21)	0.86 (2.1)	−0.04 (0.33)
Slice 12	0.2 (0.67)	−0.2 (0.35)	0.92 (2.3)	0.2 (0.67)
Slice 14	0.2 (0.58)	−0.16 (0.3)	0.68 (1.6)	−0.19 (0.29)
Slice 16	—	—	23 (NA)	−0.84 (NA)

**Table 3:** Table of tests of RMBF differences from baseline, for each sheep.

Sheep	Time	Difference	SE	*T*-value	*P* value
1	*T*1	−0.10	0.02	−6.709	<0.001
1	*T*2	−0.28	0.02	−18.003	<0.001
1	*T*3	−0.04	0.02	−2.809	0.008
1	*T*4	−0.05	0.02	−3.230	0.003
2	*T*3	3.84	0.63	6.053	<0.001
2	*T*4	0.81	0.54	1.502	0.164
3	*T*1	0.30	0.04	7.513	<0.001
3	*T*2	−0.11	0.04	−2.709	0.012
3	*T*3	−0.39	0.04	−9.690	<0.001
3	*T*4	−0.08	0.04	−1.950	0.063
4	*T*1	0.96	0.64	1.490	0.154
4	*T*2	1.29	0.64	2.009	0.060
4	*T*3	2.80	0.64	4.356	<0.001
5	*T*1	−0.34	0.10	−3.511	0.001
5	*T*2	−0.13	0.10	−1.380	0.176
5	*T*3	−0.49	0.10	−5.029	<0.001
5	*T*4	0.26	0.10	2.688	0.011

**Table 4:** Results of mixed model with a random intercept per sheep. RMBF means and confidence intervals for time.

Time	Mean	Lower	Upper
1	0.78	−0.72	2.10
2	0.78	−0.69	2.14
3	1.30	−0.13	2.66
4	0.74	−0.77	2.12

**Table 5:** Results of mixed model with a random intercept per sheep. RMBF means and confidence intervals for slices.

Slice	Mean	Lower	Upper
Medulla	1.45	0.11	2.89
Mid Brain	0.14	−1.13	1.43
Pons	0.78	−0.51	2.08
Skin	2.29	0.77	3.81
Slice 02	0.25	−0.98	1.53
Slice 04	0.32	−0.89	1.59
Slice 06	0.28	−0.91	1.59
Slice 08	0.44	−0.81	1.73
Slice 10	0.19	−1.06	1.49
Slice 12	0.34	−0.99	1.76
Slice 14	0.02	−1.30	1.43
Slice 16	−2.39	−4.76	0.36

## References

[1] Rudolph AM, Heymann MA (1967). The circulation of the fetus in utero. Methods for studying distribution of blood flow, cardiac output and organ blood flow. *Circulation research*.

[2] Makowski EL, Meschia G, Droegemueller W, Battaglia FC (1968). Measurement of umbilical arterial blood flow to the sheep placenta and fetus in utero. Distribution to cotyledons and the intercotyledonary chorion. *Circulation Research*.

[3] Magno MG, Fishman AP (1982). Origin, distribution, and blood flow of bronchial circulation in anesthetized sheep. *Journal of Applied Physiology Respiratory Environmental and Exercise Physiology*.

[4] Baile EM, Nelems JMB, Schulzer M, Pare PD (1982). Measurement of regional bronchial arterial blood flow and bronchovascular resistance in dogs. *Journal of Applied Physiology Respiratory Environmental and Exercise Physiology*.

[5] Wu C-H, Lindsey DC, Traber DL, Cross CE, Herndon DN, Kramer GC (1988). Measurement of bronchial blood flow with radioactive microspheres in awake sheep. *Journal of Applied Physiology*.

[6] Hale SL, Alker KJ, Kloner RA (1988). Evaluation of nonradioactive, colored microspheres for measurement of regional myocardial blood flow in dogs. *Circulation*.

[7] Roth JA, Greenfield AJ, Kaihara S, Wagner HN (1970). Total and regional cerebral blood flow in unanesthetized dogs. *The American journal of physiology*.

[8] Ross J (1966). Considerations regarding the technique for transseptal left heart catheterization. *Circulation*.

[9] Cheng A, Calkins H (2007). A conservative approach to performing transseptal punctures without the use of intracardiac echocardiography: stepwise approach with real-time video clips. *Journal of Cardiovascular Electrophysiology*.

[10] Hahn K, Bajwa T, Sarnoski J, Schmidt DH, Gal R (1997). Transseptal catheterization with transesophageal guidance in high risk patients. *Echocardiography*.

[11] Babaliaros VC, Green JT, Lerakis S, Lloyd M, Block PC (2008). Emerging applications for transseptal left heart catheterization old techniques for new procedures. *Journal of the American College of Cardiology*.

[12] Hung J-S, Fu M, Yeh K-H, Wu C-J, Wong P (1996). Usefulness of intracardiac echocardiography in complex transseptal catheterization during percutaneous transvenous mitral commissurotomy. *Mayo Clinic Proceedings*.

[13] Daoud EG, Kalbfleisch SJ, Hummel JD (1999). Intracardiac echocardiography to guide transseptal left heart catheterization for radiofrequency catheter ablation. *Journal of Cardiovascular Electrophysiology*.

[14] Maybauer DM, Maybauer MO, Traber LD (2006). Effects of severe smoke inhalation injury and septic shock on global hemodynamics and microvascular blood flow in sheep. *Shock*.

[15] Hodeige D, De Pauw M, Eechaute W, Weyne J, Heyndrickx GR (1999). On the validity of blood flow measurement using colored microspheres. *American Journal of Physiology. Heart and Circulatory Physiology*.

[16] Schenarts PJ, Bone HG, Traber LD, Traber DL (1996). Effect of severe smoke inhalation injury on systemic microvascular blood flow in sheep. *Shock*.

[17] Liaw PCY (2004). Endogenous protein C activation in patients with severe sepsis. *Critical care medicine*.

[18] Booke M, Westphal M, Hinder F, Traber LD, Traber DL (2003). Cerebral blood flow is not altered in sheep with *Pseudomonas aeruginosa* sepsis treated with norepinephrine or nitric oxide synthase inhibition. *Anesthesia and Analgesia*.

[19] Prinzen FW, Bassingthwaighte JB (2000). Blood flow distributions by microsphere deposition methods. *Cardiovascular Research*.

[20] Hakkinen JP, Miller MW, Smith AH, Knight DR (1995). Measurement of organ blood flow with coloured microspheres in the rat. *Cardiovascular Research*.

[21] Buckberg GD, Luck JC, Payne DB, Hoffman JI, Archie JP, Fixler DE (1971). Some sources of error in measuring regional blood flow with radioactive microspheres. *Journal of applied physiology*.

[22] Rosenberg AA, Kinsella JP, Abman SH (1995). Cerebral hemodynamics and distribution of left ventricular output during inhalation of nitric oxide. *Critical Care Medicine*.

[23] Shin C, Kinsky MP, Thomas JA, Traber DL, Kramer GC (1998). Effect of cutaneous burn injury and resuscitation on the cerebral circulation in an ovine model. *Burns*.

[24] Maybauer DM, Maybauer MO, Szabó C (2011). The peroxynitrite catalyst WW-85 improves pulmonary function in ovine septic shock. *Shock*.

[25] Maybauer MO, Maybauer DM, Fraser JF (2010). Recombinant human activated protein C attenuates cardiovascular and microcirculatory dysfunction in acute lung injury and septic shock. *Critical Care*.

[26] Yuan X-Q, Prough DS, Smith TL, Dewitt DS (1988). The effects of traumatic brain injury on regional cerebral blood flow in rats. *Journal of Neurotrauma*.

[27] Walter B, Bauer R, Krug A, Derfuss T, Traichel F, Sommer N (2002). Simultaneous measurement of local cortical blood flow and tissue oxygen saturation by Near infra-red Laser Doppler flowmetry and remission spectroscopy in the pig brain. *Acta Neurochirurgica, Supplement*.

