# Supplementary material for: Intracardiac Echocardiography Guided Transeptal Catheter Injection of Microspheres for Assessment of Cerebral Microcirculation in Experimental Models
Source: Cardiol Res Pract. 2013 Sep 11;2013:595838. doi: 10.1155/2013/595838 (PMC3786547; doi:10.1155/2013/595838)
Supplement: Supplementary file 1 — Figure 1: Results of RMBF over time by sheep and slice. Figure 2: Differences from baseline over time by sheep and slice. Figure 3: Differences from baseline over time by sheep and tissue. [file 595838.f1.pdf]

Appendix

Results of RMBF over time by sheep and slice:

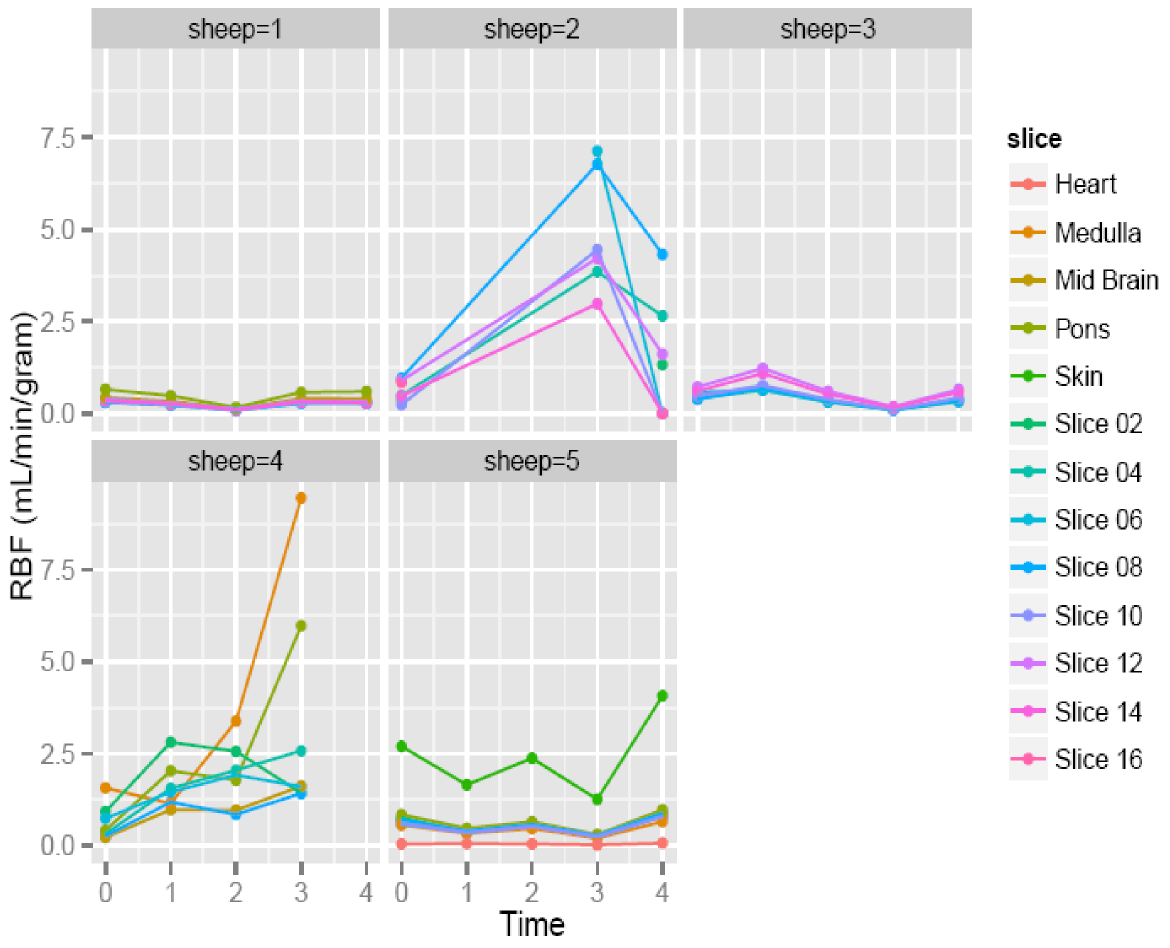

Stable RMBF over time for subject 1, 3 and 5. No Microspheres found in Subject 2 for Times 1 and 2. No microspheres found for time 4 in subject 4. Skin perfusion from subject 5 demonstrating significant variability.

Differences from baseline over time by sheep and slice.

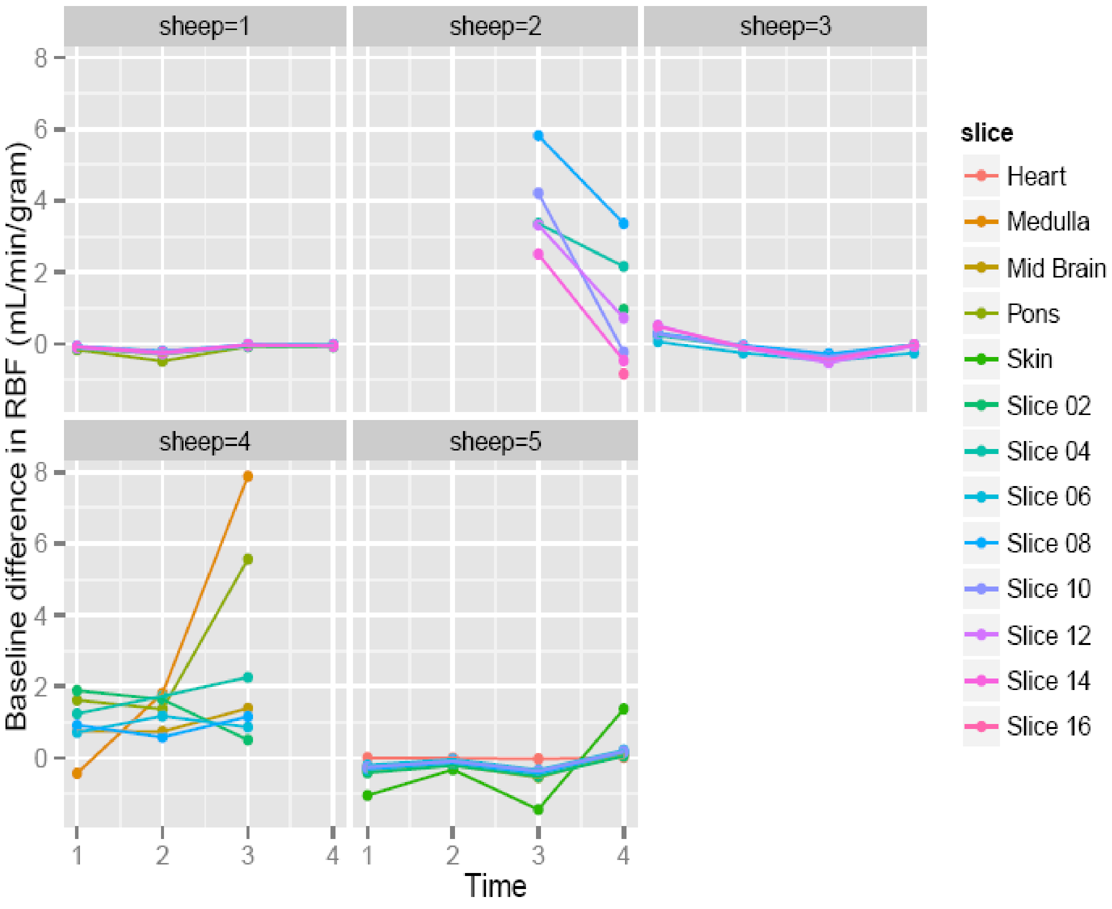

Stable RMBF over time for subject 1, 3 and 5. No Microspheres found in Subject 2 for Times 1 and 2. No microspheres found for time 4 in subject 4. Skin perfusion from subject 5 demonstrating significant variability.

Differences from baseline over time by sheep and tissue.

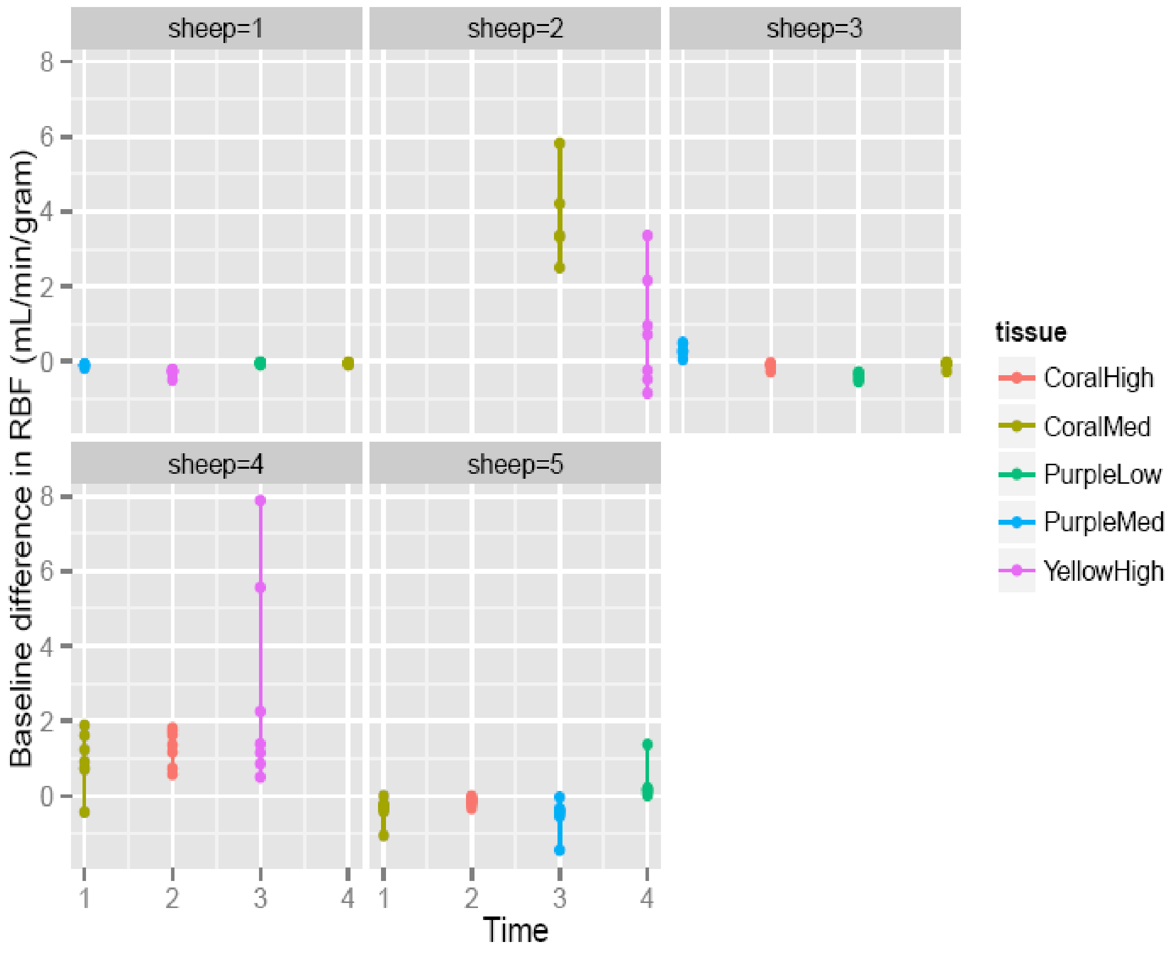

Stable RMBF over time for subject 1, 3 and 5. No Microspheres found in Subject 2 for Times 1 and 2. No microspheres found for time 4 in subject 4. Skin perfusion from subject 5 demonstrating significant variability.
